# User Personas for eHealth Regarding the Self-Management of Depressive Symptoms in People Living With HIV: Mixed Methods Study

**DOI:** 10.2196/56289

**Published:** 2025-02-17

**Authors:** Ting Zhao, Chulei Tang, Jun Ma, Huang Yan, Xinyi Su, Xueyuan Zhong, Honghong Wang

**Affiliations:** 1 Xiangya School of Nursing Central South University Changsha China; 2 School of Nursing Nanjing Medical University Nanjing China; 3 Nursing Department The Third Xiangya Hospital Central South University Changsha China

**Keywords:** HIV, depressive symptoms, depression, self-management, eHealth, personas

## Abstract

**Background:**

eHealth has enormous potential to support the self-management of depressive symptoms in people living with HIV. However, a lack of personalization is an important barrier to user engagement with eHealth. According to goal-directed design, personalized eHealth requires the identification of user personas before concrete design to understand the goals and needs of different users.

**Objective:**

This study aimed to identify user personas for eHealth regarding the self-management of depressive symptoms in people living with HIV and explore the goals and needs of different user personas for future eHealth.

**Methods:**

We used an explanatory sequential mixed methods design at the First Hospital of Changsha City, Hunan Province, China, from April to October 2022. In the quantitative phase, 572 people living with HIV completed validated questionnaires with questions related to demographics, self-efficacy, self-management abilities of depressive symptoms, and eHealth literacy. Latent profile analysis was performed to identify different user personas. In the qualitative phase, 43 one-to-one semistructured interviews across different user personas were conducted, transcribed verbatim, and analyzed using conventional content analysis. The findings from both phases were integrated during the interpretation phase.

**Results:**

Three types of user personas could be identified, including “high-level self-managers” (254/572, 44.4%), “medium-level self-managers” (283/572, 49.5%), and “low-level self-managers” (35/572, 6.1%). High-level self-managers had relatively high levels of self-efficacy, self-management abilities of depressive symptoms, and eHealth literacy. High-level self-managers had a positive attitude toward using eHealth for the self-management of depressive symptoms and desired access to self-management support for depressive symptoms from eHealth with high usability. Medium-level self-managers had relatively medium levels of self-efficacy, self-management abilities of depressive symptoms, and eHealth literacy. Medium-level self-managers felt burdened by using eHealth for the self-management of depressive symptoms and preferred to access self-management support for HIV from eHealth with privacy. Low-level self-managers had relatively low levels of self-efficacy, self-management abilities of depressive symptoms, and eHealth literacy. Low-level self-managers had an acceptable attitude toward using eHealth for the self-management of depressive symptoms and desired access to professional guidance from eHealth with privacy and no cost (“free of charge”).

**Conclusions:**

The 3 user personas shed light on the possibility of personalized eHealth to support the self-management of depressive symptoms in different people living with HIV. Further research is needed to examine the generalizability of the user personas across study sites.

## Introduction

### Background

People living with HIV disproportionately experience depressive symptoms. A previous evidence-based study estimated that the global prevalence rate of depression in people living with HIV was approximately 22% to 44% [1], which usually predicts increased sexual risk behavior, poor adherence to antiretroviral therapy, accelerated disease progression, and increased suicidal ideation [2-5].

Self-management plays a crucial role in preventing and reducing depressive symptoms in people living with HIV, and the processes include learning skills, assessing resources, and coping with the illness [6]. However, although a large number of effective resources are available for the self-management of depressive symptoms in people living with HIV, not many seek or receive these [7,8], mainly due to limited access to care, extra financial or commute burdens, and perceived stigma [9-12].

Fortunately, eHealth offers an easily accessible, flexible, potentially cost-effective, and highly anonymous option for the self-management of depressive symptoms in people living with HIV [12-14]. Since the COVID-19 pandemic, eHealth has been greatly recognized for providing better access to mental health services and is being increasingly used as a critical component of normal mental health practices [15-17]. Several existing systematic reviews have demonstrated the effectiveness of eHealth for depressive symptoms in people living with HIV [6,18,19]. Taken together, eHealth has enormous potential to support the self-management of depressive symptoms in people living with HIV [13,20].

It is worth noting, however, that users generally have various goals and needs regarding the use of eHealth [21]. Users may prefer to have an opportunity to select personalized eHealth that can be tailored to their goals and needs, and yet in practice, they always feel “depersonalized” by eHealth and perceive that developers often fail to understand their needs, which can demotivate them when engaging with such resources or even cause them to give up [22-25]. An emerging systematic review found that a lack of personalization is an important barrier to user engagement with eHealth in the mental health domain [26]. Future eHealth needs to encourage user involvement in the development phase to better understand and meet the goals and needs of various people living with HIV for the self-management of depressive symptoms, thereby promoting the adoption and sustained use of future eHealth [27].

Goal-directed design is a user research–based eHealth design method, which requires identifying user personas before concrete design to understand potential or actual user goals and needs (ie, expectations of an end condition) [28]. In this regard, user personas are detailed composite user archetypes that represent different behavior patterns and the goals and needs associated with them, providing a powerful communication tool for developers to better understand different types of users [28]. Previous evidence has indicated that user persona–tailored eHealth could increase user engagement and satisfaction with online user experience [29,30], which holds promise for the adoption and sustained use of eHealth.

According to goal-directed design, user personas can be hypothesized based on demographic variables, domain expertise, and technical expertise. Demographic variables are the most basic factors to help differentiate different types of users. We included age, gender, education, employment status, monthly household income, and health condition as the specific elements of demographic variables based on a literature review [26,31,32]. Domain expertise refers to user proficiency in a specialized subject area relevant to a product. In the area of the self-management of depressive symptoms, patients are empowered to take responsibility for the day-to-day management of their depressive symptoms [33]. However, effective self-management of depressive symptoms requires an individual to have the ability to monitor depressive symptoms and take actions necessary to maintain a satisfactory quality of life [34]. Meanwhile, self-efficacy, which refers to the belief in one’s ability to take specific actions to achieve desired outcomes [35], is closely related to effective self-management of depressive symptoms because self-efficacy, as an important motivational construct, can affect one’s choice of activities, effort, persistence, and achievement in the self-management of depressive symptoms [36]. In other words, both the self-management abilities of depressive symptoms and self-efficacy are indispensable for the desired performance in domain expertise [36,37]. We thus proposed these 2 elements as the specific elements of domain expertise. Lastly, in the eHealth area, technical expertise refers to user proficiency in eHealth technology. We thus defined technical expertise as the ability to seek, find, understand, and appraise health information from electronic sources and apply the knowledge gained to address or solve a health problem, namely eHealth literacy [38].

### Objectives

To inform the design of future eHealth, this study aimed to identify user personas for eHealth regarding the self-management of depressive symptoms in people living with HIV and explore the goals and needs of different user personas for future eHealth. We propose the following hypotheses:

- Hypothesis 1: There will be different user personas for eHealth regarding the self-management of depressive symptoms in people living with HIV.
- Hypothesis 2: Different user personas will reflect different people living with HIV, their characteristics, and their goals and needs for future eHealth.

## Methods

### Study Design

We undertook an explanatory sequential mixed methods study [39]. First, we conducted a quantitative survey to provide a preliminary understanding of user personas for eHealth regarding the self-management of depressive symptoms in people living with HIV. Then, we collected qualitative data through semistructured interviews to explain these user personas in more depth and explore the goals and needs of different user personas for future eHealth. Lastly, the quantitative and qualitative findings were integrated during the interpretation phase.

This study has been reported in line with the STROBE (Strengthening the Reporting of Observational Studies in Epidemiology) checklist for cross-sectional studies [40], the SRQR (Standards for Reporting Qualitative Research) checklist [41], and the GRAMMS (Good Reporting of a Mixed Methods Study) checklist [42].

### Quantitative Phase

#### Setting and Sampling

We recruited a consecutive sample of people living with HIV on arrival at the HIV clinic of the First Hospital of Changsha City, Hunan Province, China, from April 2022 to October 2022. Hunan Province is located in south-central China, with a population of 53,030 people living with HIV at the end of October 2022, similar to the national-level prevalence [43]. The First Hospital of Changsha City is a large designated tertiary hospital for HIV diagnosis and treatment in Hunan Province, which follows about 9000 HIV/AIDS patients, with an annual outpatient volume of about 30,000 visits [44]. Individuals were eligible if they met the following criteria: (1) were aged 18 years or older, (2) had been diagnosed with HIV infection (as confirmed by a diagnosis report), and (3) voluntarily participated in this survey after providing informed consent. Individuals were excluded if they had cognitive or audiovisual impairment assessed by clinicians. We consecutively screened people living with HIV for eligibility to participate in this survey until a sufficient sample size was achieved. Meanwhile, some potential participants were referred by medical social workers. Overall, a total of 600 participants were involved in the survey, and 592 returned completed surveys. A final sample of 572 participants was included for analysis after removing responses with over 10% missing data.

#### Variables and Measures

Demographic variables included age (years), gender (male=0, female=1), educational level (high school or below=0, higher education or above=1), employment status (unemployed=0, employed=1), monthly household income (<10,000 RMB=0, ≥10,000 RMB=1; a currency exchange rate of RMB 1=US $0.1375 is applicable), and health condition (including comorbidities [no=0, yes=1], latest CD4 count [≥200 cells/mm^3^=0, <200 cells/mm^3^=1], viral load [not “target not detected (TND)” status=0, TND status=1], and severity of depressive symptoms), with these categories determined by reference to previous literature [26,45-48]. We used the 9-item Patient Health Questionnaire (PHQ-9) to assess the severity of depressive symptoms in the past 2 weeks [49], with a total score range from 0 to 27. Scores of 5, 10, 15, and 20 represent mild, moderate, moderately severe, and severe depression, respectively.

The self-management abilities of depressive symptoms were assessed using the 9-item Chinese version of the Depression-Specific Self-Management Questionnaire (DSSM; a 5-point Likert scale), which has a total score ranging from 9 to 45, with higher scores indicating higher levels of self-management abilities of depressive symptoms [50]. Self-efficacy was assessed using the Self-efficacy for Managing Chronic Disease Scale (SEMCDS; a 10-point scale) [51]. The scale has 6 items, and the total score can range from 6 to 60, with higher scores indicating higher levels of self-efficacy. eHealth literacy was assessed using the 8-item Chinese version of the eHealth Literacy Scale (eHEALS; a 5-point Likert scale), which has a total score ranging from 8 to 40, with higher scores indicating higher levels of eHealth literacy [52,53]. All Cronbach α values were above the cutoff value of .70, indicating good internal consistency [54].

#### Data Collection

Before the formal survey, we pretested the questionnaire and adjusted the questionnaire wording as necessary to ensure comprehensibility and readability. We trained 4 investigators (graduate nursing students) to distribute and collect questionnaires using a traditional pen-and-paper method. Each participant completed the questionnaire themselves after the investigator demonstrated how to fill in responses. For those who could not read or write, the investigator explained the meaning of each item and completed the questionnaire according to the participant’s responses. At the end of the survey, participants were invited to participate in a follow-up qualitative interview. Those who expressed an interest were asked to leave their WeChat contact details.

#### Data Analysis

All returned questionnaires were double-entered in EpiData software (version 3.1; EpiData Association) to ensure accuracy and integrity. Statistical analysis consisted of 3 parts. First, we conducted descriptive statistics to understand the characteristics of the participants.

Second, we used latent profile analysis (LPA) to identify the latent profiles of user personas among people living with HIV based on responses to the following indicators: the self-management abilities of depressive symptoms, self-efficacy, and eHealth literacy. Participants with similar response patterns were classified into the same profile. To ensure adequate accuracy of the classification, the recommended minimum sample size for LPA studies is at least 500 [55,56], and therefore, the current size of 572 is sufficient.

We used a maximum likelihood estimation with robust standard errors (MLR) to identify the participants’ latent profiles [57]. To avoid local maxima, we used 7000 random sets of starting values for the initial stage, 500 iterations for each random start, and the 200 best solutions retained for final-stage optimizations. To identify the optimal number of latent profiles, we evaluated 1- to 5-profile models based on the statistical fit, parsimony, and substantive interpretability [58]. We considered a combination of fit indices, including Akaike Information Criterion (AIC), Bayesian Information Criterion (BIC), sample size–adjusted BIC (saBIC), and entropy [57]. The model with lower AIC, BIC, and saBIC values had a better fit, while the model with an entropy value closer to 1 had a higher classification accuracy. We also used the adjusted Lo-Mendel-Rubin likelihood ratio test (aLMRT) and bootstrap likelihood ratio test (BLRT) to evaluate model fit [57]. A statistically significant aLMRT or BLRT value indicates that the model with *k* profiles outperforms that with *k-1* profiles. In addition, the percentage of individuals in the smallest profile was set at 5% of the total sample to include at least sufficient individuals (n=30-60) to support the generalizability of the LPA [59,60].

Third, we used the automatic 3-step approach to model the covariates after identifying the optimal number of latent profiles. Specifically, we used the R3STEP command, which assigns individuals to their most likely profiles, to conduct a series of multinomial logistic regressions to examine the effects of covariates on the profile membership [61,62]. This procedure is the recommended method as it accounts for the measurement error in profile classification while modeling the effects of covariates on profile membership [60,61]. All demographic variables were introduced as covariates. In the R3STEP procedure, we used multiple imputation to impute missing values for covariates to minimize bias. A total of 27 imputations were used according to a rule of thumb (ie, the number of imputations should be at least equal to the percentage of incomplete cases) [63]. All statistical analyses were conducted in Mplus software (version 8.4) [64], and a *P* value of <.05 was considered statistically significant.

### Qualitative Phase

#### Sampling

The qualitative phase was conducted using a qualitative descriptive design based on the naturalistic inquiry [65], which is especially appropriate for mixed methods research to provide a rich straight description of the facts from the participants’ points of view [66]. From August to October 2022, we recruited 43 participants for the qualitative interviews via a WeChat invitation. We used stratified purposeful sampling to recruit heterogeneous participants with different ages, genders, educational levels, employment statuses, monthly household incomes, or health conditions for each user persona from the quantitative sample [67]. Individuals were eligible if they had completed the quantitative survey of this study and agreed to participate in the qualitative interview. The sample size was determined by the principle of data saturation (ie, no new analytical information arose anymore) [68].

#### Procedure and Analysis

Three qualitatively trained investigators (female graduate nursing students) used a semistructured interview guide to conduct in-depth interviews. The interview guide consisted of open-ended questions informed by the quantitative findings, a group discussion, and a preliminary pilot study with 5 patients, including the following key topics: (1) how do they self-manage depressive symptoms? (2) what are their attitudes toward using eHealth for the self-management of depressive symptoms? (3) what are their goals and needs toward using eHealth for the self-management of depressive symptoms? Interviews took place in a private room at the HIV clinic, at the participant’s home, or in another quiet location, either face-to-face or by telephone, whichever was convenient for participants. The length of interviews ranged from 20 to 82 minutes. No one else was present except the participant and the interviewer. The interviewers had no dependency relationship with any participants.

Each interview was audio-recorded, immediately transcribed verbatim, and member-checked to ensure accuracy. Field notes (eg, verbal and nonverbal cues, “off-the-record” texts) were also used to enrich our results further. Two trained investigators repeatedly reviewed the interview transcripts, immersed themselves in these to obtain a sense of the whole interview, and then independently conducted conventional content analysis using NVivo software (version 12; Lumivero) to identify patterns and categories in an inductive approach. An initial codebook was developed after the first 5 interviews and was iteratively refined in the following interviews until a consensus was reached. Disagreements were resolved through discussion among a 5-member review team having multi-disciplinary backgrounds in nursing, HIV, and mental health.

### Mixed Methods Data Integration

We used a mixed methods approach to identify user personas to provide a deeper insight and understanding than either approach [39]. As noted, the quantitative findings informed the sampling frame and interview guide of the following qualitative phase (ie, integration at the methods level) [69]. Second, we used a statistics-by-themes joint display as a visual means to integrate the main quantitative and qualitative findings at the analytic and interpretation levels [70], with the following three possible outcomes: (1) confirmation, where the quantitative and qualitative findings confirm each other; (2) expansion, where the quantitative and qualitative findings diverge and expand insights into user personas; and (3) discordance, where the qualitative and quantitative findings are inconsistent, incongruous, contradictory, or conflicting, or disagree with each other [69]. The integration of quantitative and qualitative findings identified key targets for the design of future eHealth. Multimedia Appendix 1 shows the flow of the overall study.

### Ethical Considerations

The study was performed in accordance with the Declaration of Helsinki and received ethics approval from the Institutional Review Board of Xiangya School of Nursing, Central South University (number: E202210). All participants provided verbal informed consent before formal participation in the quantitative or qualitative phase. Pseudonyms were used during data collection, storage, and presentation to maintain anonymization and deidentification (ie, linked only to the study ID). No compensation was provided to participants during the quantitative phase. Upon completing the qualitative phase, participants received US $6.85 for their participation.

## Results

### Quantitative Findings

#### Participant Characteristics

A total of 572 valid questionnaires were included in this study (see Multimedia Appendix 2). Participants were between 18 and 72 years old, with a median age of 29 (IQR 25-34.8) years. The majority of participants were male (541/572, 94.6%), had higher education or above (452/572, 79.0%), were employed (472/572, 82.5%), and had a monthly household income of ≥10,000 RMB (≥10,000 Chinese Yuan; 390/572, 68.2%). In addition, 22.2% (127/572) of the participants had comorbidities, but most had a latest CD4 count of ≥200 cells/mm^3^ (463/572, 80.9%) and an undetectable HIV viral load (368/572, 64.3%). The PHQ-9 scores of the participants ranged from 0 to 27, with a median score of 6 (IQR 3-11), and 64.3% (368/572) had mild or more depressive symptoms. Detailed descriptive statistics are shown in Table 1.

**Table 1:** Participant characteristics in the quantitative phase (N=572).

Variable		Total (N=572)	High-level self-managers (n=254)	Medium-level self-managers (n=283)	Low-level self-managers (n=35)
Age (years), median (IQR)		29 (25-34.8)	29 (25-35)	28 (25-34)	31 (25-37)
**Gender, n (%)**					
	Male	541 (94.6)	240 (94.5)	270 (95.4)	31 (88.6)
	Female	31 (5.4)	14 (5.5)	13 (4.6)	4 (11.4)
**Education, n (%)**					
	High school or below	120 (21.0)	44 (17.3)	68 (24.0)	8 (22.9)
	Higher education or above	452 (79.0)	210 (82.7)	215 (76.0)	27 (77.1)
**Employment status, n (%)**					
	Unemployed	100 (17.5)	31 (12.2)	57 (20.1)	12 (34.3)
	Employed	472 (82.5)	223 (87.8)	226 (79.9)	23 (65.7)
**Monthly household income (RMB^a^), n (%)**					
	<10,000^b^	157 (27.4)	55 (21.7)	85 (30.0)	17 (48.6)
	≥10,000	390 (68.2)	189 (74.4)	184 (65.0)	17 (48.6)
	Missing	25 (4.4)	10 (3.9)	14 (4.9)	1 (2.9)
**Presence of comorbidities, n (%)**					
	No	443 (77.4)	212 (83.5)	208 (73.5)	23 (65.7)
	Yes	127 (22.2)	41 (16.1)	74 (26.1)	12 (34.3)
	Missing	2 (0.3)	1 (0.4)	1 (0.4)	0 (0.00)
**Latest CD4 count (cells/mm^3^), n (%)**					
	≥200	463 (80.9)	203 (79.9)	234 (82.7)	26 (74.3)
	<200^c^	31 (5.4)	17 (6.7)	9 (3.2)	5 (14.3)
	Missing	78 (13.6)	34 (13.4)	40 (14.1)	4 (11.4)
**Viral load, n (%)**					
	Not TND^d^ status	93 (16.3)	39 (15.4)	47 (16.6)	7 (20.0)
	TND status	368 (64.3)	167 (65.7)	180 (63.6)	21 (60.0)
	Missing	111 (19.4)	48 (18.9)	56 (19.8)	7 (20.0)
Severity of depressive symptoms, median (IQR)		6 (3-11)	3 (1-6)	8 (6-12)	19 (12-25)
Self-efficacy, mean (SE)		41.4 (0.5)	51.4 (0.7)	35.9 (1.1)	16.9 (2.3)
DSSM^e^, mean (SE)		31.4 (0.2)	34.7 (0.4)	29.4 (0.3)	24.7 (1.0)
eHealth literacy, mean (SE)		29.5 (0.3)	31.9 (0.5)	27.9 (0.4)	24.8 (1.3)

^a^RMB: Renminbi (Chinese yuan).

^b^RMB 1=US $0.1375.

^c^Latest CD4 count <200 cells/mm^3^ indicates advanced HIV disease [46].

^d^TND: target not detected.

^e^DSSM: self-management abilities of depressive symptoms.

#### Model Selection

Table 2 presents fit statistics for 1- to 5-profile structures. As the number of profiles increased, the solutions provided lower AIC, BIC, and saBIC values and statistically significant BLRT values. However, the smallest profile of the 4- or 5-profile solutions contained less than 5% of the total sample. Considering the principle of substantive interpretability, we did not examine these 2 profile solutions further, as these profiles may be spurious [60]. We finally retained the 3-profile solution as the optimal solution because it exhibited lower AIC, BIC, and saBIC values and a higher entropy value than the 2-profile solution, with statistically significant aLMRT and BLRT values, indicating a significant improvement in fit statistics compared to the 2-profile solution. Meanwhile, the smallest profile of the 3-profile solution contained more than 5% of the total sample (35/572, 6.1%), which is the minimum reference standard to support the generalizability of the LPA [59,60]. Average posterior probabilities for individuals assigned to each profile in this solution were 0.898, 0.879, and 0.928, respectively, with all values greater than 0.70, indicating well-separated profiles [71].

**Table 2:** Fit statistics for 1- to 5-profile structures (N=572).

Number of profiles	AIC^a^	BIC^b^	saBIC^c^	Entropy	aLMRT^d^ (*P*)	BLRT^e^ (*P*)	Latent profile proportion
1	4881.797	4907.892	4888.844	—^f^	—	—	1.000
2	4667.982	4711.473	4679.728	0.627	<.001	<.001	0.579/0.421
3	4615.057	4675.944	4631.501	0.762	.003	<.001	0.495/0.444/0.061
4	4591.966	4670.251	4613.109	0.736	.84	<.001	0.521/0.364/0.096/0.019
5	4529.099	4624.780	4554.940	0.790	.008	<.001	0.540/0.308/0.108/0.033/0.010

^a^AIC: Akaike Information Criterion.

^b^BIC: Bayesian Information Criterion.

^c^saBIC: sample-size adjusted BIC.

^d^aLMRT: adjusted Lo-Mendel-Rubin likelihood ratio test.

^e^BLRT: bootstrap likelihood ratio test.

^f^Not applicable.

#### Profile Characteristics

**Figure 1:**
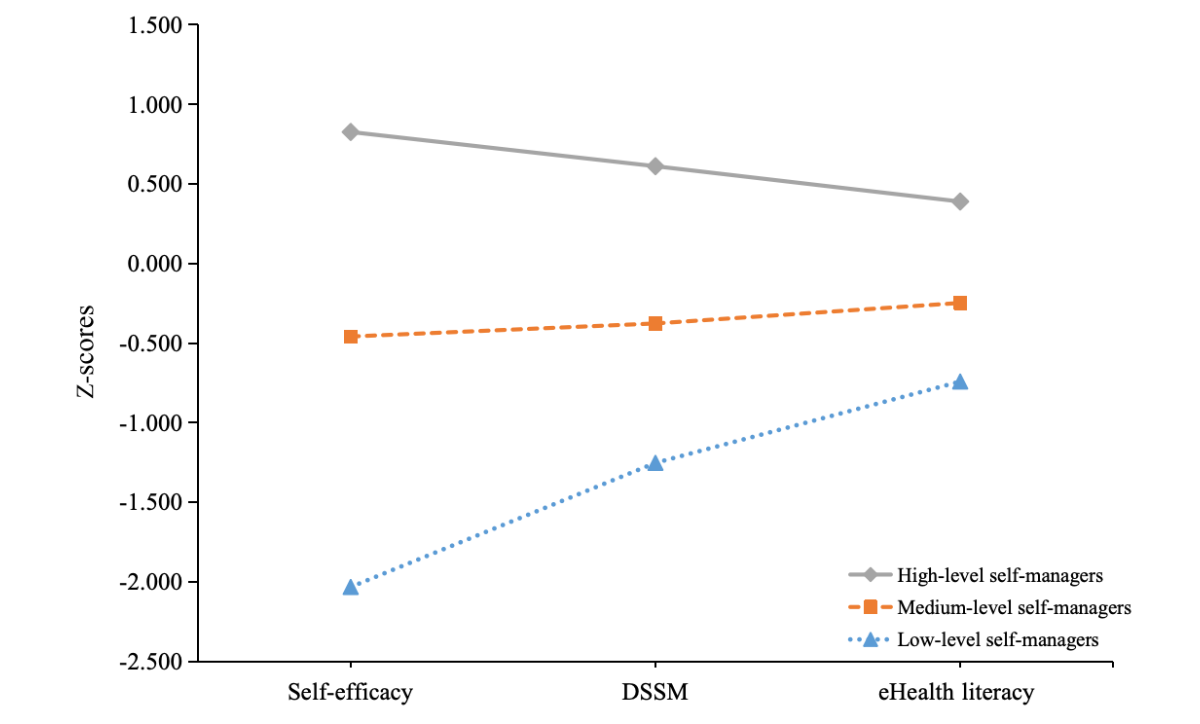
Three-profile structure based on z-scores (N=572). DSSM: self-management abilities of depressive symptoms.

Table 1 displays the estimated means and SEs for the 3 indicators in each profile. Profile 1 (254/572, 44.4%; *high-level self-managers*) was characterized by relatively higher self-efficacy (mean 51.4, SE 0.7), self-management abilities of depressive symptoms (mean 34.7, SE 0.4), and eHealth literacy (mean 31.9, SE 0.5). Profile 2 (283/572, 49.5%; *medium-level self-managers*) was characterized by relatively medium self-efficacy (mean 35.9, SE 1.1), self-management abilities of depressive symptoms (mean 29.4, SE 0.3), and eHealth literacy (mean 27.9, SE 0.4). Profile 3 (35/572, 6.1%; *low-level self-managers*) was characterized by relatively lower self-efficacy (mean 16.9, SE 2.3), self-management abilities of depressive symptoms (mean 24.7, SE 1.0), and eHealth literacy (mean 24.8, SE 1.3). Figure 1 presents a graphical representation of the 3 profiles based on the z-scores.

#### Three-Step Approach

The 3-step results for the covariates are shown in Multimedia Appendix 3. Compared with *high-level self-managers*, we found that *medium-level self-managers* and *low-level self-managers* were more likely to have comorbidities and more severe depressive symptoms. Additionally, we found that *low-level self-managers* were more likely to have more severe depressive symptoms than *medium-level self-managers*.

### Qualitative Findings

#### Interview Sample Characteristics

In total, 43 of 572 individuals who completed quantitative surveys volunteered to participate in semistructured interviews, including 23 *high-level self-managers*, 17 *medium-level self-managers*, and 3 *low-level self-managers*. The sample consisted of 37 males and 6 females, with a median age of 29 years (IQR 25.5-37; range 19-70 years). A total of 30 participants had used eHealth for the self-management of depressive symptoms. See Multimedia Appendix 4 for more details.

The findings are summarized by characteristics for the self-management of depressive symptoms, attitudes toward using eHealth, and goals and needs toward using eHealth. See Multimedia Appendix 5 for representative quotes.

#### Characteristics for the Self-Management of Depressive Symptoms

High-level self-managers were *particularly proactive* in the self-management of depressive symptoms, such as proactively seeking relevant information and social support. High-level self-managers believed that their emotional management plays a critical role in their health conditions and can, in the worst case, be devastating or fatal.

Medium-level self-managers were *proactive* in the self-management of depressive symptoms, but most used distraction techniques, such as playing on their phones. Medium-level self-managers believed that health conditions were the leading cause of their depressive symptoms and thus emphasized the moderating effects of health conditions on their depressive symptoms.

Low-level self-managers were *negative* about the self-management of depressive symptoms. Low-level self-managers often did not know how to make themselves better.

#### Attitudes Toward Using eHealth

High-level self-managers had a *positive* attitude toward using eHealth for the self-management of depressive symptoms. High-level self-managers often proactively used eHealth to manage their depressive symptoms, including searching for health information, following up on the latest news about HIV, and seeking peer advice.

Medium-level self-managers felt *burdened* toward using eHealth for the self-management of depressive symptoms. Medium-level self-managers usually did not use eHealth to manage their depressive symptoms to avoid potential burdens caused by frequent pushes, such as anxiety, feeling down, or feeling different from others.

Low-level self-managers felt it was *acceptable* to use eHealth for the self-management of depressive symptoms, especially for online guidance from professionals.

#### Goals and Needs Toward Using eHealth

High-level self-managers desired *access to self-management support for depressive symptoms* from eHealth, including (1) psychological support: psychological counseling, psychological information, peer support, and referral, and (2) HIV-related support: HIV-related information and counseling. Meanwhile, high-level self-managers desired eHealth to have *high usability*, including privacy, a user-friendly interface, personalized content, a positive and healthy atmosphere, and increased fun.

Medium-level self-managers focused on *access to self-management support for HIV* from eHealth, including HIV-related information and counseling, and emphasized the *privacy* of eHealth.

Low-level self-managers desired *access to guidance from professionals*, either psychological or HIV-related. Meanwhile, low-level self-managers desired eHealth with *privacy* and *no cost (free of charge)*.

### Integrated Findings

The main quantitative and qualitative findings were integrated into a joint display. See Multimedia Appendix 5 for more details.

High-level self-managers had relatively high levels of self-efficacy, self-management abilities of depressive symptoms, and eHealth literacy. High-level self-managers had a positive attitude toward using eHealth for the self-management of depressive symptoms and desired access to self-management support for depressive symptoms from eHealth with high usability.

Medium-level self-managers had relatively medium levels of self-efficacy, self-management abilities of depressive symptoms, and eHealth literacy. Medium-level self-managers emphasized the moderating effects of health conditions on their depressive symptoms and felt burdened by using eHealth for the self-management of depressive symptoms. As a result, medium-level self-managers preferred to access self-management support for HIV from eHealth with privacy.

Low-level self-managers had relatively low levels of self-efficacy, self-management abilities of depressive symptoms, and eHealth literacy. Low-level self-managers had an acceptable attitude toward using eHealth for the self-management of depressive symptoms and desired access to professional guidance from eHealth with privacy and no cost (free of charge).

## Discussion

### Principal Findings and Comparison With Prior Work

This mixed methods study identified 3 types of user personas for eHealth regarding the self-management of depressive symptoms in people living with HIV, including high-level self-managers, medium-level self-managers, and low-level self-managers. The different user personas reflect different people living with HIV, their characteristics, and their goals and needs for future eHealth. Therefore, developers may need to consider a tailored perspective in the design of eHealth for different user personas.

High-level self-managers were characterized by relatively high levels of self-efficacy, self-management abilities of depressive symptoms, and eHealth literacy, with minimal depressive symptoms. People living with HIV in this user persona attached great importance to the impact of emotional management on their health condition, and thus, they were particularly proactive in the self-management of depressive symptoms. This phenomenon may be explained by the fact that people with high self-efficacy and self-management abilities tend to see difficulties as challenges to overcome rather than to avoid, so they act with more proactive coping efforts in threatening situations [37]. In addition, people with fewer depressive symptoms tend to perceive more benefits and fewer barriers to taking effective measures to manage their depressive symptoms, so they are more likely to engage in self-management proactively [72]. Moreover, considering their positive attitudes toward eHealth, it is unsurprising that high-level self-managers desired access to more self-management support for depressive symptoms from eHealth, which could make them feel more empowered to control their health [25,26]. Notably, high-level self-managers desired eHealth with high usability, including privacy, a user-friendly interface, personalized content, a positive and healthy atmosphere, and increased fun, similar to the findings in previous research [25,26,73]. These findings may be explained by their relatively high levels of eHealth literacy [25], suggesting that future eHealth for high-level self-managers could be designed from a multi-dimensional perspective to provide an ideal interactive feel.

Medium-level self-managers were characterized by relatively medium levels of self-efficacy, self-management abilities of depressive symptoms, and eHealth literacy, with comorbidities and more severe depressive symptoms. People living with HIV in this user persona were proactive in the self-management of depressive symptoms, whereas most tended to use distraction techniques and placed more emphasis on the moderating effects of health conditions on their depressive symptoms. Accordingly, they preferred to access self-management support for HIV from eHealth. This phenomenon may be partly because people with comorbidities may experience more complexity in self-management, such as additional physical or psychological strain [74,75]. As a result, they tend to struggle with self-management and feel less confident to do so [76-78]. Therefore, it makes sense that medium-level self-managers desired access to self-management support for HIV from eHealth. In addition, this phenomenon may be explained by the traditional cultural beliefs and the stigma against depression in Chinese society [79-81], where people tend to keep their mental discomfort to themselves or refer to mental discomfort as physical symptoms to maintain their “face” (dignity, reputation, and public image), which may prevent them from seeking help for mental health [82,83]. Previous research also confirmed that many people living with HIV in China hardly ever use mental health services, including those with persistent depression [8]. Similar phenomena have been found in other geographical areas, such as Europe, South Asia, and South-East Asia [84]. These findings suggest that future eHealth should not only meet the goals and needs of medium-level self-managers but also improve their recognition in the self-management of depressive symptoms to promote behavioral change. Finally, our findings indicated that medium-level self-managers tended to feel burdened by eHealth resources with frequent push notifications, and thus, they used such resources less often, which is in agreement with previous meta-ethnographic findings [25]. These findings suggest that future eHealth for medium-level self-managers should be designed to promote their use of eHealth in the right amount according to the interactive principle of “Not too much. Not too little. Just right.” [85].

Low-level self-managers were characterized by relatively low levels of self-efficacy, self-management abilities of depressive symptoms, and eHealth literacy, with comorbidities and the most severe depressive symptoms. People living with HIV in this user persona were negative about the self-management of depressive symptoms. One possible explanation is that people with low self-efficacy tend to regard their efforts as futile in the face of difficulties and thus quickly give up trying, which, combined with their low self-management abilities, makes it difficult for them to achieve the desired performance in the self-management of depressive symptoms [37]. Another possible explanation is that people with severe depressive symptoms usually experience diminished interest in doing things, loss of energy, reduced ability to think or concentrate, or indecisiveness, and as a result, they often have difficulty in proactive self-management [72,86,87]. However, our findings indicated that low-level self-managers had an acceptable attitude toward eHealth resources and desired access to professional guidance from eHealth with privacy and no cost (free of charge). This finding suggests that future eHealth for low-level self-managers could be designed as a connection point for 2-way communication with an interactive feeling of “minimizing privacy risks and maximizing public benefits.”

Notably, all 3 types of user personas emphasized the privacy of eHealth, such as invitation codes and non-HIV–related names, which may be attributed to HIV-related stigma and discrimination. Although antiretroviral therapy has transformed HIV infection into a manageable chronic health condition [88], people living with HIV worldwide continue to face significant stigma and discrimination at the individual, interpersonal, community, and societal levels, including avoidance, gossip, verbal or physical abuse, social rejection, denial of health or social services, denial or loss of employment or education opportunities, or even arrest [89,90]. As a result, people living with HIV often value privacy to avoid unwanted disclosures and try to live a “normal” life [91,92]. Previous research has also emphasized the privacy concerns regarding eHealth platforms for HIV management, even in developed countries [92-94]. These findings suggest that future eHealth should be designed with adequate consideration of regulatory, privacy, and security protocols, regardless of user personas.

### Strengths and Limitations

To our knowledge, this is the first study to construct user personas for eHealth regarding the self-management of depressive symptoms in people living with HIV. We constructed this powerful communication tool based on goal-directed design, providing an effective way for user involvement in the development phase of eHealth [28,95]. In addition, this study used an explanatory sequential mixed methods design, which integrated quantitative and qualitative findings to provide a more in-depth exploration of user personas. Our findings may be of use to gain a better understanding of intended end users and inform the design of future eHealth.

However, this study has several limitations. First, participants were recruited from a single site in the metropolitan area, and thus, our findings might not be generalizable to other areas of China. However, the site is a large designated tertiary hospital for HIV diagnosis and treatment, with a good representation of the diverse patient population in the survey area. Therefore, it can still be assumed that our findings reflect a social trend. Second, most participants were young males, which might be explained by the difficult access to vulnerable people living with HIV (eg, females) [96]. Therefore, the findings should be interpreted with caution. It would be helpful to include more vulnerable people living with HIV in future research to reflect their unique needs. Third, we recruited only 3 low-level self-managers for qualitative interviews, which might have contributed to potential bias. This may be due to the small total number of low-level self-managers and the fact that low-level self-managers usually experienced the most severe depressive symptoms, with diminished interest in interviews. However, we endeavored to ensure the heterogeneity of the participants by stratified purposeful sampling as much as possible, and the qualitative data revealed no new analytical information, so we presumed data saturation. Finally, as the study did not collect data over time, we did not investigate the dynamics of user personas. Future research can examine this vital area to gain more information to guide the design of future eHealth.

### Implications for Practice

Our findings provide a practical communication aid, namely user personas, for interdisciplinary collaborations between health researchers and developers, which contributes to the integration of the “art of caring” and the “art of design” in future eHealth to provide personalized support for the self-management of depressive symptoms in people living with HIV [28,97]. Specifically, user personas could help frontline health researchers better play the role of requirement analysts during interdisciplinary collaborations to bring the user voice to the forefront in a clear and easily understandable way, thereby helping developers to keep different types of users and their goals and needs in mind during the early stages of development and continuously throughout the evaluation and implementation phases [95,97]. Furthermore, user personas could help elicit specific advice from health care providers, regulators, and investors by making the intended end users more tangible, which can contribute to addressing broader system and policy constraints [98].

### Conclusions

According to goal-directed design, we identified 3 types of user personas for eHealth regarding the self-management of depressive symptoms in people living with HIV: high-level self-managers, medium-level self-managers, and low-level self-managers. The 3 user personas shed light on the possibility of personalized eHealth to support the self-management of depressive symptoms in different people living with HIV. Further research is needed to examine the generalizability of the user personas across study sites.

## Appendix

Multimedia Appendix 1 Flowchart for the explanatory sequential mixed methods design.

Multimedia Appendix 2 Participant flow throughout the study.

Multimedia Appendix 3 Three-step results for covariates in the quantitative phase (n=572).

Multimedia Appendix 4 Participant characteristics in the qualitative phase (n=43).

Multimedia Appendix 5 Joint display of the main quantitative and qualitative findings.

Multimedia Appendix 6 GRAMMS (Good Reporting of A Mixed Methods Study) checklist.

Multimedia Appendix 7 STROBE (Strengthening the Reporting of Observational Studies in Epidemiology) checklist for cross-sectional studies.

Multimedia Appendix 8 SRQR (Standards for Reporting Qualitative Research) checklist.

## Abbreviations

AIC: Akaike Information Criterion
aLMRT: adjusted Lo-Mendel-Rubin likelihood ratio test
BIC: Bayesian Information Criterion
BLRT: bootstrap likelihood ratio test
LPA: latent profile analysis
PHQ-9: 9-item Patient Health Questionnaire
saBIC: sample size–adjusted Bayesian Information Criterion
TND: target not detected

## Data Availability

The datasets generated during or analyzed during this study are available from the corresponding author upon reasonable request.
