# Supplementary material for: User Personas for eHealth Regarding the Self-Management of Depressive Symptoms in People Living With HIV: Mixed Methods Study
Source: J Med Internet Res. 2025 Feb 17;27:e56289. doi: 10.2196/56289 (PMC11888057; doi:10.2196/56289)
Supplement: Multimedia Appendix 2 [file jmir_v27i1e56289_app2.doc]

**Multimedia Appendix 2.** Participant flow throughout the study.
