# Supplementary material for: User Personas for eHealth Regarding the Self-Management of Depressive Symptoms in People Living With HIV: Mixed Methods Study
Source: J Med Internet Res. 2025 Feb 17;27:e56289. doi: 10.2196/56289 (PMC11888057; doi:10.2196/56289)
Supplement: Multimedia Appendix 3 [file jmir_v27i1e56289_app3.doc]

**Multimedia Appendix 3.** Three-step results for covariates in the quantitative phase (n=572)^a^.

| Profiles  comparison | Age | Female | EDU^e^ | Employed | Monthly household income^f^ | Having comorbidities | CD_4_ count <200 cells/mm^3^ | TND status^g^ | Severity of depressive symptoms |
| --- | --- | --- | --- | --- | --- | --- | --- | --- | --- |
| C2^c^ vs C1^b^ | 0.01 | -0.21 | -0.37 | -0.37 | -0.73 | 1.21^*^ | -0.98 | -0.09 | 0.51^**^ |
| C3^d^ vs C1^b^ | 0.02 | 0.90 | 0.20 | -0.57 | -1.35 | 1.60^*^ | -0.98 | -0.32 | 0.71^**^ |
| C2^c^ vs C3^d^ | -0.01 | -1.11 | -0.58 | 0.20 | 0.62 | -0.39 | 0.01 | 0.23 | -0.20^**^ |

^a^Values in the table are estimates from the R3STEP logistic regression analyses. Positive values indicate that the particular covariate makes an individual more likely to be classified as the first latent profile than the second latent profile; negative values indicate the opposite.

^b^C1: High-level self-manager.

^c^C2: Medium-level self-manager.

^d^C3: Low-level self-manager.

^e^EDU: with a higher education or above.

^f^Monthly household income: ≥10000 RMB (Chinese yuan).

^g^TND status: target not detected (HIV viral load <20 copies/ml).

^*^*P*<.05, ^**^*P*<.01.
