# Supplementary material for: User Personas for eHealth Regarding the Self-Management of Depressive Symptoms in People Living With HIV: Mixed Methods Study
Source: J Med Internet Res. 2025 Feb 17;27:e56289. doi: 10.2196/56289 (PMC11888057; doi:10.2196/56289)
Supplement: Multimedia Appendix 4 [file jmir_v27i1e56289_app4.doc]

**Multimedia Appendix 4. Participant characteristics in the qualitative phase (n=43).**

| Participant  ID | Age (years) | Gender | Education | Employment  status | Monthly household income  (Chinese yuan) | Having comorbidities | Latest  CD_4_ count (cells/mm^3^) | Viral load^a^ | The severity of depression | Experiences with eHealth^b^ | Group |
| --- | --- | --- | --- | --- | --- | --- | --- | --- | --- | --- | --- |
| A1 | 23 | Male | Higher education or above | Yes | <10000 | No | ≥200 | - | Moderate | No | High-level self-manager |
| A2 | 32 | Male | Higher education or above | Yes | ≥10000 | No | - | TND status | Mild | Yes | High-level self-manager |
| A3 | 26 | Male | Higher education or above | Yes | ≥10000 | No | ≥200 | - | Mild | Yes | High-level self-manager |
| A4 | 38 | Female | Higher education or above | Yes | ≥10000 | No | ≥200 | TND status | Mild | No | High-level self-manager |
| A5 | 28 | Male | Higher education or above | Yes | ≥10000 | No | ≥200 | TND status | Mild | No | High-level self-manager |
| A6 | 30 | Male | Higher education or above | Yes | ≥10000 | No | ≥200 | Not TND status | Mild | Yes | High-level self-manager |
| A7 | 33 | Male | Higher education or above | Yes | <10000 | Yes | ≥200 | - | Moderately severe | Yes | High-level self-manager |
| A8 | 29 | Male | Higher education or above | Yes | ≥10000 | No | ≥200 | TND status | Minimal | Yes | High-level self-manager |
| A9 | 27 | Male | Higher education or above | Yes | ≥10000 | No | ≥200 | TND status | Mild | Yes | High-level self-manager |
| A10 | 29 | Male | Higher education or above | Yes | ≥10000 | No | ≥200 | Not TND status | Minimal | Yes | High-level self-manager |
| A11 | 28 | Male | Higher education or above | No | <10000 | No | ≥200 | TND status | Minimal | No | High-level self-manager |
| A12 | 33 | Male | Higher education or above | Yes | ≥10000 | No | ≥200 | TND status | Mild | Yes | High-level self-manager |
| A13 | 20 | Male | Higher education or above | Yes | ≥10000 | No | ≥200 | - | Mild | Yes | High-level self-manager |
| A14 | 20 | Male | Higher education or above | Yes | <10000 | No | ≥200 | - | Mild | Yes | High-level self-manager |
| A15 | 19 | Male | Higher education or above | Yes | ≥10000 | No | <200 | Not TND status | Minimal | Yes | High-level self-manager |
| A16 | 25 | Male | Higher education or above | Yes | ≥10000 | No | ≥200 | TND status | Minimal | Yes | High-level self-manager |
| A17 | 22 | Male | Higher education or above | No | ≥10000 | Yes | ≥200 | Not TND status | Mild | No | High-level self-manager |
| A18 | 34 | Male | Higher education or above | Yes | ≥10000 | No | ≥200 | TND status | Minimal | No | High-level self-manager |
| A19 | 65 | Male | Higher education or above | No | <10000 | No | ≥200 | TND status | Minimal | Yes | High-level self-manager |
| A20 | 28 | Male | Higher education or above | Yes | - | No | ≥200 | TND status | Mild | No | High-level self-manager |

**Multimedia Appendix 4.** Continued.

| Participant ID | Age (years) | Gender | Education | Employment  status | Monthly household income  (Chinese yuan) | Having comorbidities | Latest  CD_4_ count (cells/mm^3^) | Viral load^a^ | The severity of depression | Experiences with eHealth^b^ | Group |
| --- | --- | --- | --- | --- | --- | --- | --- | --- | --- | --- | --- |
| A21 | 48 | Female | High school or below | No | <10000 | No | ≥200 | TND status | Mild | No | High-level self-manager |
| A22 | 70 | Male | High school or below | No | <10000 | No | ≥200 | TND status | Mild | Yes | High-level self-manager |
| A23 | 39 | Male | High school or below | Yes | ≥10000 | No | ≥200 | TND status | Mild | Yes | High-level self-manager |
| B1 | 21 | Male | Higher education or above | No | <10000 | No | ≥200 | - | Moderate | Yes | Medium-level self-manager |
| B2 | 26 | Male | Higher education or above | Yes | ≥10000 | Yes | ≥200 | TND status | Moderately severe | Yes | Medium-level self-manager |
| B3 | 33 | Male | Higher education or above | Yes | ≥10000 | No | ≥200 | TND status | Mild | Yes | Medium-level self-manager |
| B4 | 67 | Female | Higher education or above | No | <10000 | Yes | - | - | Moderate | No | Medium-level self-manager |
| B5 | 20 | Male | Higher education or above | Yes | ≥10000 | No | - | TND status | Mild | No | Medium-level self-manager |
| B6 | 44 | Male | Higher education or above | Yes | ≥10000 | No | ≥200 | TND status | Minimal | No | Medium-level self-manager |
| B7 | 25 | Male | Higher education or above | Yes | <10000 | Yes | ≥200 | Not TND status | Minimal | Yes | Medium-level self-manager |
| B8 | 27 | Male | Higher education or above | Yes | ≥10000 | No | ≥200 | TND status | Mild | Yes | Medium-level self-manager |
| B9 | 40 | Male | Higher education or above | No | <10000 | No | ≥200 | TND status | Moderate | Yes | Medium-level self-manager |
| B10 | 27 | Male | Higher education or above | No | <10000 | Yes | ≥200 | - | Moderate | Yes | Medium-level self-manager |
| B11 | 26 | Male | Higher education or above | Yes | ≥10000 | No | ≥200 | TND status | Mild | Yes | Medium-level self-manager |
| B12 | 32 | Male | Higher education or above | Yes | ≥10000 | Yes | ≥200 | TND status | Mild | Yes | Medium-level self-manager |
| B13 | 40 | Male | Higher education or above | Yes | ≥10000 | No | ≥200 | TND status | Mild | Yes | Medium-level self-manager |
| B14 | 23 | Male | High school or below | Yes | ≥10000 | No | ≥200 | TND status | Minimal | Yes | Medium-level self-manager |
| B15 | 40 | Female | High school or below | Yes | <10000 | No | - | TND status | Mild | No | Medium-level self-manager |
| B16 | 61 | Female | High school or below | No | - | Yes | - | - | Mild | Yes | Medium-level self-manager |
| B17 | 26 | Male | Higher education or above | No | ≥10000 | No | ≥200 | TND status | Moderately severe | Yes | Medium-level self-manager |

**Multimedia Appendix 4.** Continued.

| Participant ID | Age (years) | Gender | Education | Employment  status | Monthly household income  (Chinese yuan) | Having comorbidities | Latest  CD_4_ count (cells/mm^3^) | Viral load^a^ | The severity of depression | Experiences with eHealth^b^ | Group |
| --- | --- | --- | --- | --- | --- | --- | --- | --- | --- | --- | --- |
| C1 | 21 | Male | Higher education or above | No | <10000 | No | ≥200 | - | Moderately severe | No | Low-level self-manager |
| C2 | 36 | Male | Higher education or above | Yes | <10000 | Yes | ≥200 | TND status | Mild | No | Low-level self-manager |
| C3 | 33 | Female | Higher education or above | No | <10000 | No | ≥200 | TND status | Mild | Yes | Low-level self-manager |

^a^TND: target not detected.

^b^Experiences with eHealth regarding the self-management of depressive symptoms.
