# Supplementary material for: User Personas for eHealth Regarding the Self-Management of Depressive Symptoms in People Living With HIV: Mixed Methods Study
Source: J Med Internet Res. 2025 Feb 17;27:e56289. doi: 10.2196/56289 (PMC11888057; doi:10.2196/56289)
Supplement: Multimedia Appendix 5 [file jmir_v27i1e56289_app5.doc]

**Multimedia Appendix 5.** Joint display of the main quantitative and qualitative findings.

| User persona | Main quantitative findings | Main qualitative findings | | Integrated findings |
| --- | --- | --- | --- | --- |
|  |  | Categories | Representative quotes |  |
| High-level self-managers | **Domain expertise** | **Category 1. Characteristics for self-management of depressive symptoms** | |  |
|  | - Self-management abilities of depressive symptoms:   34.7 (0.4)^a^;   - Self-efficacy:   51.4 (0.7)^a^; | *Particularly proactive* | - a1): When I knew that this disease (HIV) might cause emotional problems, I looked for a lot of relevant information, like psychological knowledge. I also got together with some good friends for a drink or a chat, and their advice could also help you manage yourself better. When you know about these, you feel that anyway (laughs) … you can figure out how to regulate yourself pretty well. - a2): I think the disease itself may not be scary. The scariest parts include two: one is emotional management, which … may be more likely to break or kill a person than the disease itself. … If you are in a good mood, you are not sick. | - **Confirmation:**   High-level self-managers had relatively high levels of self-efficacy and self-management abilities of depressive symptoms. |
|  | **Technical expertise** | **Category 2. Attitudes toward using eHealth** | |  |
|  | - eHealth literacy:   31.9 (0.5)^a^; | *Positive* | - a3): I would search some WeChat official accounts online to help identify my depression, such as ‘Do I have depression?’ or ‘How do I manage this condition?’ - a4): I sometimes googled the latest news on this disease (HIV). When I read the information related to new medications, I thought maybe … we would be cured someday, so I did not get so depressed during that time. It is beneficial to build up mentally, so we like to know more. - a5): I like going surfing to see what state other patients are in and what advice they have. I have observed that many people take their disease seriously, do not seek death, and return to a normal state of mind. After reading all this information, it seems to me that it (HIV) is not a big deal. | - **Confirmation:**   High-level self-managers had a relatively high level of eHealth literacy.   - **Expansion:**   High-level self-managers had a positive attitude toward using eHealth for self-management of depressive symptoms. |

**Multimedia Appendix 5.** Continued.

| User persona | Main quantitative findings | Main qualitative findings | | Integrated findings |
| --- | --- | --- | --- | --- |
|  |  | Categories | Representative quotes |  |
| High-level self-managers |  | **Category 3.**  **Goals and** **needs toward using eHealth** | |  |
|  |  | - *Access to self-management support for depressive symptoms* - *High usability* | - a6): The main thing should be psychological counseling to help me determine if I have any signs of depression lately (laughs) or anything else. ..., I'd also like to have some information to inform people about mental health. For example, what is the clinical manifestation of depression, or how to do self-observation? …, I desire to talk to some fellow patients and encourage each other, which would also have a good effect on the treatment. …, I can use this platform to directly find an offline hospital or relevant psychological counseling facilities to seek medical attention offline. - a7): I would like it to be updated with news about HIV treatment in real time so that we can feel at ease. …. It would be nice to have a manual platform for us to ask questions directly. Because even if we know that many things may be okay, we still need psychological comfort, like whether we can share towels with our families. In fact, if you are never clear about these things, it can put you into a tight one. - a8): First, your interface can't be too fancy, or it will make people feel very uncomfortable. …, You can find some information quickly and easily. Your interface should at least make people know what this part is for at a glance, and then I can do what I want to do in this part. - a9): The name should not be too obvious, as well as the logo (laughs). …, It should never be obvious to anyone else for privacy. | - **Expansion:**   High-level self-managers desired access to self-management support for depressive symptoms from eHealth with high usability. |

**Multimedia Appendix 5.** Continued.

| User persona | Main quantitative findings | Main qualitative findings | | Integrated findings |
| --- | --- | --- | --- | --- |
|  |  | Categories | Representative quotes |  |
| High-level self-managers |  | **Category 3.**  **Goals and** **needs toward using eHealth** | |  |
|  |  |  | - a10): When we log in for the first time, we can do some simple interest selections or a psychological test, and then the platform will recommend some appropriate content to us. - A11): It should have a positive, healthy atmosphere on the platform, such as positive words or mental health tips every day. I desire this platform will have a positive impact on my work and life. - a12): If it simply shows you the information without interpretation or some interesting points, I probably will not read it. …, I think there could be some entertainment, such as interactive games, or people could speak in a live room, or some small games that help patients integrate into a group, which could alleviate many psychological problems. |  |

**Multimedia Appendix 5. Continued.**

| User persona | Main quantitative findings | Main qualitative findings | | Integrated findings |
| --- | --- | --- | --- | --- |
|  |  | Categories | Representative quotes |  |
| Medium-level self-managers | **Domain expertise** | **Category 1. Characteristics for self-management of depressive symptoms** | |  |
|  | - Self-management abilities of depressive symptoms:   29.4 (0.3)^a^;   - Self-efficacy:   35.9 (1.1)^a^; | *Proactive* | - b1): When I am unhappy, I usually distract myself by thinking about happy things or doing something I enjoy. I feel like playing on my phone would stop me from thinking about those unhappy things just as soon. - b2): I read about depression, but I feel like that probably did not help me because you are certainly not in a good mood if you are not in good health. I believe health conditions associated with this disease (HIV) are the main reason for depression. It is better to focus on our health. | - **Confirmation:**   Medium-level self-managers had relatively medium levels of self-efficacy and self-management abilities of depressive symptoms.   - **Expansion:**   Medium-level self-managers emphasized the moderating effects of health conditions on their depressive symptoms. |
|  | **Technical expertise** | **Category 2. Attitudes toward using eHealth** | |  |
|  | - eHealth literacy:   27.9 (0.4)^a^; | *Burdened* | - b3): If there is a push for something I am interested in, I might read it, but I usually do not click on it because I am worried about how often Big Data will push it on me. I generally do not feel uncomfortable, but if it is pushed to me too often, it might make me feel anxious instead. I would subconsciously feel different from others. This, in turn, would make me down. | - **Confirmation:**   Medium-level self-managers had a relatively medium level of eHealth literacy.   - **Expansion:**   Medium-level self-managers felt burdened by using eHealth for self-management of depressive symptoms. |

**Multimedia Appendix 5.** Continued.

| User persona | Main quantitative findings | Main qualitative findings | | Integrated findings |
| --- | --- | --- | --- | --- |
|  |  | Categories | Representative quotes |  |
| Medium-level self-managers |  | **Category 3.**  **Goals and** **needs toward using eHealth** | |  |
|  |  | - *Access to self-management support for HIV* - *Privacy* | - b4): I desire to have a manual Q&A. For example, when the first test results came out, we saw a row of abnormal data marked in red, which made us pessimistic. So, I would like someone to tell us which data to focus on and which abnormal data we need to seek immediate medical attention. I would also like to browse information about treatments, new medications, and so on. …, I think the main thing related to our emotions is the effectiveness of the treatment for this disease (HIV). You should first develop this core module that patients urgently need and then consider the emotional modules. - b5): If it's exclusively for people like us, the first thing you need to do is to protect our privacy. Not everyone can use the platform. People need the invitation code. | - **Expansion:**   Medium-level self-managers desired access to self-management support for HIV from eHealth with privacy. |

**Multimedia Appendix 5.** Continued.

| User persona | Main quantitative findings | Main qualitative findings | | Integrated findings |
| --- | --- | --- | --- | --- |
|  |  | Categories | Representative quotes |  |
| Low-level self-managers | **Domain expertise** | **Category 1. Characteristics for self-management of depressive symptoms** | |  |
|  | - Self-management abilities of depressive symptoms:   24.7 (1.0)^a^;   - Self-efficacy:   16.9 (2.3)^a^; | *Negative* | - c1): I feel especially miserable, …, sometimes I think about happy things, but ... I do not feel like it is helpful. I told my parents I was depressed, but they thought I was crazy. …, I do not know how to make myself better. I have the idea to record things that happen to me every day, but I do not have the perseverance to do that. I feel like there is nothing happy to record. | - **Confirmation:**   Low-level self-managers had relatively low levels of self-efficacy and self-management abilities of depressive symptoms. |
|  | **Technical expertise** | **Category 2. Attitudes toward using eHealth** | |  |
|  | - eHealth literacy:   24.8 (1.3)^a^; | *Acceptable* | - c2): I tend to utilize online resources. I've googled: Why am I unhappy? What happens when you get depressed? But I do not think these helped much. … I don't know where to find such a (health) platform, or else I would use it. I can accept online guidance from professionals. I hope to solve my problems and not keep thinking about these unhappy things. | - **Confirmation:**   Low-level self-managers had a relatively low level of eHealth literacy.   - **Expansion:**   Low-level self-managers were acceptable to use eHealth for self-management of depressive symptoms. |

**Multimedia Appendix 5.** Continued.

| User persona | Main quantitative findings | Main qualitative findings | | Integrated findings |
| --- | --- | --- | --- | --- |
|  |  | Categories | Representative quotes |  |
| Low-level self-managers |  | **Category 3.**  **Goals and** **needs toward using eHealth** | |  |
|  |  | - *Access to* *guidance from professionals* - *Privacy and no cost (free of charge)* | - c3): I would like to consult professionals about something I don't understand or worry about, as I consulted some general knowledge related to HIV the other day. After understanding these, I will feel better. Otherwise, I scare myself or get a knot in my heart. ..., If I had this app, I would first seek a professional to solve my psychological problems. I'm afraid of getting along with my family or those close to me. What should I do? - c4): I desire this platform to be like a WeChat official account. If it's an app with an HIV-related name, I'm worried that as soon as I open the interface, someone else will see it. - c5): I desire the consultation to be free because many hospitals now charge a lot for online consultations, tens or hundreds of dollars at a time. It is charged by time, like how much for a minute. Maybe the first minute or the first picture is free, but after that, there is a charge. If you consult a specialist, the cost will be higher, and the time may run out before you finish your questions. | - **Expansion:**   Low-level self-managers desired access to professionals’ guidance from eHealth with privacy and no cost (free of charge). |

^a^means (standard errors, SEs).
