# Supplementary material for: User Personas for eHealth Regarding the Self-Management of Depressive Symptoms in People Living With HIV: Mixed Methods Study
Source: J Med Internet Res. 2025 Feb 17;27:e56289. doi: 10.2196/56289 (PMC11888057; doi:10.2196/56289)
Supplement: Multimedia Appendix 6 [file jmir_v27i1e56289_app6.pdf]

Good reporting of a mixed-methods study (GRAMMS) checklist

Guideline	Page information
1. Describe the justification for using a mixed methods approach to the research question	4-5,9
2. Describe the design in terms of the purpose, priority and sequence of methods	4-5
3. Describe each method in terms of sampling, data collection and analysis	5-9
4. Describe where integration has occurred, how it has occurred and who has participated in it	9
5. Describe any limitation of one method associated with the present of the other method	19
6. Describe any insights gained from mixing or integrating methods	15-20

Reference: O'Cathain A, Murphy E, Nicholl J. The quality of mixed methods studies in health services research. J Health Serv Res Policy. 2008;13: 92-98.
